# Supplementary material for: Association of Fortification with Human Milk versus Bovine Milk-Based Fortifiers on Short-Term Outcomes in Preterm Infants—A Meta-Analysis
Source: Nutrients. 2024 Mar 21;16(6):910. doi: 10.3390/nu16060910 (PMC10975992; doi:10.3390/nu16060910)
Supplement: Supplementary file 1 [file nutrients-16-00910-s001.zip › nutrients-2914791-supplementary/File S1.pdf]

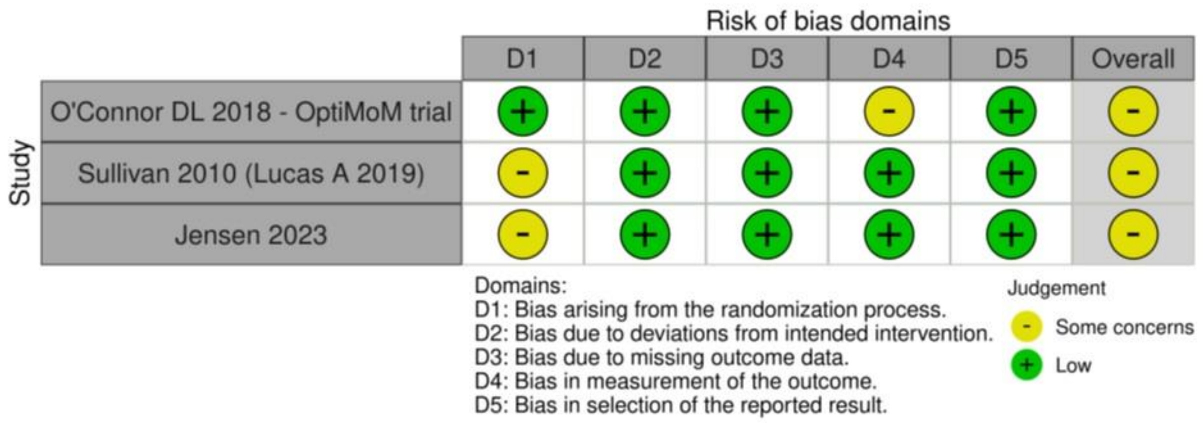

Figure S1: Risk of Bias (RoB) [1–3].

Supplimentary File S1: Search strategy—Effects of exclusively using human milk with HMFs versus BMFs

Search performed on: 31.08.2023

PubMed:
(((("Infant, Newborn"[Mesh] OR "Infant, Extremely Premature"[Mesh] OR "Infant, Premature"[Mesh] OR "Infant, Very Low Birth Weight"[Mesh] OR "Gestational Age"[Mesh] OR "Infant, Extremely Low Birth Weight"[Mesh] OR neonat*[tiab] OR infant*[tiab] OR newborn*[tiab] OR gestation*[tiab] OR "pre-term"[tiab] OR "birth weight"[tiab] OR neonat*[OT] OR infant*[OT] OR newborn*[OT] OR gestation*[OT] OR "pre-term"[OT] OR "birth weight"[OT])) AND ((("Infant Formula"[Mesh] OR "Milk, Human"[Mesh] OR "Breast Feeding"[Mesh:NoExp] OR "Milk"[Mesh:NoExp] OR "Food, Fortified"[Mesh] OR "Milk Banks"[Mesh] OR milk[tiab] OR formula[tiab] OR feeding[tiab] OR Milk[OT] OR formula[OT] OR feeding[OT])) AND ((fortif*[tiab] OR exclusiv*[tiab] OR predominant*[tiab] OR supplement*[tiab] OR fortif*[OT] OR exclusiv*[OT] OR predominant*[OT] OR supplement*[OT]))) AND (("Enterocolitis, Necrotizing"[Mesh] OR "Retinopathy of Prematurity"[Mesh] OR "Bronchopulmonary Dysplasia"[Mesh] OR "Mortality"[Mesh] OR "Infant Mortality"[Mesh] OR "Morbidity"[Mesh] OR "necrotizing enterocolitis"[tiab] OR "necrotising enterocolitis"[tiab] OR NEC[tiab] OR "retinopathy of prematurity"[tiab] OR "bronchopulmonary dysplasia"[tiab] OR "Mortality"[tiab] OR "Morbidity"[tiab] OR "necrotizing enterocolitis"[OT] OR "necrotising enterocolitis"[OT] OR NEC[OT] OR "retinopathy of prematurity"[OT] OR "bronchopulmonary dysplasia"[OT] OR "Mortality"[OT] OR "Morbidity"[OT]))))

Filters: from 2015–2023
PubMed: English: 1413, All languages: 1449

Table S1: Baseline characteristics comparing infants fed human milk vs bovine milk-derived fortifier

Parameter	HM-HMF	HM-BMF
**Jensen et. al- NORDIC [1]**		
Number of subjects	115	113
Sex (female)	54/115	52/113
Chorioamnionitis	27/115	16/113
Apgar at 5 min	7 (median IQR 5-8)	7 (median IQR 5-8)
Gestation (weeks)	25.5 (SD 1.5)	25.7 (SD 1.6)
Birthweight (grams)	793 (SD 212)	787 (SD 207)
**OptiMoM trial [2]**		
Number of subjects	64	63
Sex (female)	39/64	34/63
Race (non-European)	37/64	44/63
Antenatal steroids	56/64	56/63
Apgar at 5 min	7.4 (SD 2.1)	7.3 (SD 2.3)
Gestation (weeks)	27.9 (SD 2.7)	27.5 (SD 2.3)
Birthweight (grams)	887 (SD 208)	889 (SD 196)
SGA at birth	13/64 (20%)	16/63 (25%)
**Sullivan trial re-analysis [3]**		
Number of subjects	82	32
Sex(female)	47/82 (57.3%)	15/32 (46.9%)
Race (black)	16/82 (19.5%)	3/32 (9.4%)
Antenatal steroids	15/82 (18.3%)	6/32 (18.8%)
Apgar <7	8/82 (9.8%)	6/32 (18.8%)
Gestation (weeks)	27.3 ± 2.2	27.1 ± 1.8
Birthweight (grams)	937 ± 199	938 ± 190
SGA at birth	10/82 (12.2%)	3/32 (9.4%)
**Assad study re-analysis [4]**		
Number of subjects	87	127
Sex (female)	34/87 (39%)	64/127 (50%)
Race (black)	53 (61%)	85/127 (67%)
Gestation (weeks)	27.7 (SD 2.7)	28.3 (SD 2.8)

HM-HMF: Human milk with human milk fortifier; HM-BMF: Human milk with bovine milk fortifier; SGA: Small for gestational age;

Table S2: Outcomes comparing infants fed human milk vs. bovine milk-derived fortifier

Parameter	HM-HMF	HM-BMF	RR	P-value
Total number subjects	348	333		
**Jensen et al.- NORDIC [1]**				
Number of subjects	115	113		
NEC (Bell stage II-III)	8 (7%)	9 (8%)	1.1	0.77
NEC surgical	4 (4%)	4 (4%)	1	1.00
Death	7 (6%)	13 (12%)	2	0.15
Culture-proven sepsis	33 (29%)	28 (25%)	0.9	0.50
BPD	60/108 (56%)	66/102 (65%)	1.2	0.18
ROP III-V	29/113 (26%)	25/110 (23%)	0.9	0.61
Mortality and morbidity index (MMI)	78 (68%)	85 (75%)	1.1	0.22
Time full enteral feeds, days	10 (median IQR 8-15)	10 (median IQR 8-13)		0.27
**OptiMoM trial [2]**				
Number of subjects	64	61		
ROP (severe)	1/62 (1.6%)	6/59	6.4	0.04
Late onset sepsis	8/64 (13%)	14/61 (23%)	1.8	0.07
Death	3/64 (4.7%)	4 (6.6%)	1.4	0.65
BPD	16/64 (25%)	18/61 (30%)	1.2	0.73
NEC (Bells stage II or greater)	3/64 (4.7%)	3/61 (4.9%)	1.0	0.95
NEC all stages	3/64	6/61	2.1	0.27
Feeds withheld 12 h (FW12h)	17/64 (27%)	20/61 (23%)	1.6	0.19
Positive morbidity index	23/64 (36%)	30/61 (49%)	1.4	0.07
**Sullivan RCT- subgroup reanalysis [3]**				
Number of subjects	82	32		
NEC (Bells Stage II or greater)	3/82 (3.7%)	5 (15.6%)	4.2	0.04
NEC surgery or death	3/82 (3.7%)	6/32 (18.8%)	5.1	0.01
Death only	3/82 (3.7%)	4/32 (12.5%)	3.4	0.10
Proven sepsis	20/84 (29.3%)	11/32 (34.4%)	0.6	0.45
BPD	24/84 (29.3%)	11/32 (34.4%)	1.2	0.60
ROP (grade 3 or 4)	6/84 (7.3%)	2/32 (6.3%)	0.9	1.0
**Assad study- subgroup reanalysis [4]**				
Number of subjects	87	127		
NEC (Bell stage II or greater)	1/87 (1.1%)	11/127 (8.7%)	7.5	0.02
ROP	11/87 (14%)	40/127 (32%)	2.5	0.001
BPD	13/87 (15%)	30/127 (24%)	1.6	0.20
PDA	7/87 (8%)	28/127 (22%)	2.7	0.007
Feeds withheld 24 h (FW24h)	5/87 (6%)	43/127 (34%)	5.9	0.001
Late-onset sepsis	11/87 (13%)	20/127 (16%)	1.3	0.66

HM-HMF: Human milk with human milk fortifier; HM-BMF: Human milk with bovine milk fortifier; NEC: Necrotizing enterocolitis; BPD: Bronchopulmonary dysplasia; ROP: Retinopathy of prematurity.

References

1. Bach Jensen, G.a.D., Magnus and Ahlsson, Fredrik and Elfvin, Anders and Naver, Lars and Abrahamsson, Thomas. Effect Of Human Milk-Based Fortification in Extremely Preterm Infants Fed Exclusively with Breast Milk: A Randomised Controlled Trial. Available at SSRN: . Available online: https://papers.ssrn.com/sol3/papers.cfm?abstract_id=4529245 (accessed on
2. O'Connor, D.L.; Kiss, A.; Tomlinson, C.; Bando, N.; Bayliss, A.; Campbell, D.M.; Daneman, A.; Francis, J.; Kotsopoulos, K.; Shah, P.S.; et al. Nutrient enrichment of human milk with human and bovine milk-based fortifiers for infants born weighing <1250 g: a randomized clinical trial. *Am J Clin Nutr* **2018**, *108*, 108-116, doi:10.1093/ajcn/nqy067.

3. Lucas, A.; Boscardin, J.; Abrams, S.A. Preterm Infants Fed Cow's Milk-Derived Fortifier Had Adverse Outcomes Despite a Base Diet of Only Mother's Own Milk. *Breastfeed Med* **2020**, *15*, 297-303, doi:10.1089/bfm.2019.0133.
4. Lucas, A.; Assad, M.; Sherman, J.; Boscardin, J.; Abrams, S. Safety of Cow's Milk-Derived Fortifiers Used with an All-Human Milk Base Diet in Very Low Birthweight Preterm Infants. *Neonatology Today* **2020**, *15*, 3-13, doi:10.51362/neonatology.today/20207157313.
